# The evaluation of biological indices to assess the condition of hillslope seep wetlands in the Tsitsa River Catchment, South Africa

**DOI:** 10.1371/journal.pone.0251370

**Published:** 2021-05-18

**Authors:** Notiswa Libala, Carolyn G. Palmer, Oghenekaro Nelson Odume

**Affiliations:** Unilever Centre for Environmental Water Quality, Institute for Water Research, Rhodes University, Grahamstown, South Africa; Feroze Gandhi Degree College, INDIA

## Abstract

The increase in the degradation of wetlands globally has highlighted the need to assess their ecological condition. Hillslope seep wetlands are among the least studied wetland types, yet they the most vulnerable because of their small size and steep slopes. Human pressure and the vulnerable nature of these wetlands requires wetland assessment tools to assess their condition. This study sought to evaluate the performance of the Floristic Quality Assessment Index for all species (FQAIall), the FQAI for dominant species (FQAIdom), and the Floristic Assessment Quotient for Wetlands (FAQWet) in response to the Anthropogenic Activity Index (AAI) and WET-Health in eleven hillslope seep wetlands and used these indices to assess the degree and intensity of disturbance. Vegetation samples were collected in summer 2016 and winter 2017. All assessment indices, FQAIall, FQAIdom, FAQWet and WET-Health, showed that hillslope seep wetlands were impacted by human activities. FQAIall showed the strongest response to AAI in winter, while FAQWet showed the strongest response to WET-Health. To the best of our knowledge, researchers in South Africa have used only WET-Health to assess wetland condition, and this is the first study to assess the condition of hillslope seep wetlands using a combination of indices (FQAIall, FQAIdom, FAQWet, and WET-Health). Overall, the findings of this study suggest that FQAIall and FAQWet are potentially better tools for assessing the biological condition of hillslope seep wetlands in South Africa.

## Introduction

Wetlands play a crucial role in maintaining the functioning of aquatic ecosystems in the landscape [1]. They are among the most utilised ecosystems, providing valuable services such as water for domestic use, grazing for livestock, land for cultivation, and fibre for crafts and construction [2].

Despite their importance, wetlands are under severe threat [3,4,5] with an estimated 50% of the world’s wetlands lost to agricultural activities [6]. Human pressure on wetland ecosystems has necessitated the development of a range of wetland assessment techniques and approaches such as the Floristic Quality Assessment Index (FQAI) [7,8], Wetland Index Value (WIV) [9], WET-Health [10], and the Floristic Assessment Quotient for Wetlands FAQWet [11]. These techniques have been developed to assess the health and condition of wetlands to inform management decisions.

The Floristic Quality Assessment Index (FQAI) is one of the most widely used wetland assessment tools in the United States of America (USA) [8,12,13,14] and is used for estimating the biological condition of wetlands based on the overall conservatism of the species assemblage [15]. The FQAI employs a numeric quality rating and a coefficient of conservatism (CC) to indicate the affinity of plant species to a particular habitat, or the species’ tolerance of disturbances [16]. The CC ranges from 0 to 10, where high CC scores (9–10) indicate that such plants have a high fidelity to particular habitat types and are less tolerant of disturbances, and plants with low CC scores (0–3) are those that are found in a wide variety of habitat types and disturbed regimes [12] The resulting list of CC scores is used to calculate indices such as the FQAI and a mean CC [17].

Because FQAI requires a comprehensive list of species with coefficients of conservatism that are not readily available in most countries [18 and 12] modified FQAI, using only dominant species. When FQAI (dominant) and FQAI (all species) were compared, no significant differences were observed in the correlations of these two versions of FQAI with human pressure [18]. The FQAI (dominant) is useful because most users are able to identify common wetland plants [12], but its use may result in homogenisation of plant lists, making the tool less sensitive to anthropogenic disturbance [12].

For the same reason–that comprehensive records of species coefficients of conservatism are unavailable for most regions [11], developed the Floristic Assessment Quotient for Wetlands Index (FAQWet index) which incorporates the wetland indicator status and the native status of plant species and serves as an alternative to FQAI where coefficients of conservatism are unavailable [11]. The FAQWet index includes information on the presence of exotic species not included in the original FQAI because [11] argue that exotic species may have negative consequences for wetland condition, regardless of regional conservatism of native species present.

In South Africa, the most widely used wetland assessment tool is WET-Health [10]. The WET-Health tool was designed for assessing the ecological condition of wetlands based on the impacts of human-induced stressors on hydro-geomorphic processes and vegetation responses [19]. It uses the Present Ecological State (PES) of the hydrology, geomorphology and vegetation cover of a wetland, and the anticipated future trajectory of change. In assessing the ecological condition, the differences between FQAI, FAQWet and WET-Health are: i) the level of detail at which the vegetation is assessed, which is higher for FQAI and FAQWet than for WET-Health, ii) the degree to which the method relies on the professional judgement of the assessor in scoring the vegetation, which is high for WET-Health, and iii) FQAI and FAQWet are response indices, while WET-Health is a stressor index.

Indices developed to date are used to assess general wetland conditions, but their performance for assessing hillslope seep wetlands has not been widely tested. Hillslope seep wetlands differ from other types of wetland in that i) they are small, ranging in size between 0.05 to 1.2 ha, and therefore extremely vulnerable to disturbances; ii) they depend heavily on groundwater or on sub-surface water inputs, which can easily be influenced by seasonality; iii) they are located on steep slopes, further exacerbating their potential vulnerability to pressure; iv) their evergreen nature within the context of the broader catchment makes them attractive for all-year grazing by cattle and sheep and are thus subject to intense pressure. Given the uniqueness of hillslope seep wetlands, several different vegetation-based indices are available for assessing the ecological condition of wetlands, but in South Africa to date there has been very little examination of how these relate to each other and to the level of anthropogenic disturbance. Also, there has been little investigation into how the assessments of these indices differ seasonally. The objectives of this study, then, were i) to evaluate the performances of FQAIall, FQAIdom, and FAQWet in assessing the ecological health of the selected hillslope seep wetlands by regressing them against anthropogenic disturbance activity (AAI) and WET-Health. Both AAI and WET-Health methods are strongly based on describing stressors and they rely on a high degree of subjective opinion on the part of the assessor in assigning the scores; ii) to test the variation of indices between winter and summer seasons, iii) to assess the spatial-temporal redundancy between FQAIall and FQAIdom to ascertain whether they can be used interchangeably in the context of hillslope seep wetlands.

### Study area description

**Fig 1:**
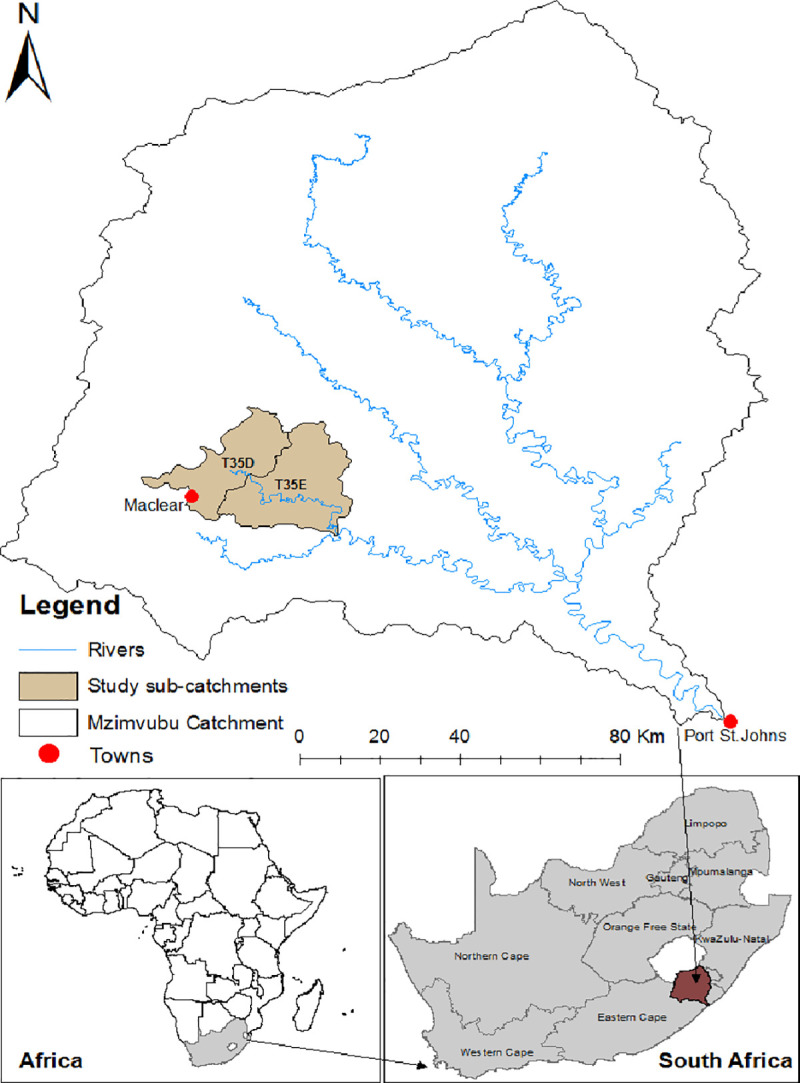
Locality map of quaternary catchments T35D and T35E in the Tsitsa River catchment within the Mzimvubu catchment, Eastern Cape, South Africa.

The study was conducted in two quaternary catchments (T35D and T35E) situated in the Tsitsa River catchment, in the Eastern Cape of South Africa (Fig 1). A quaternary catchment is defined as a fourth-order catchment in a hierarchal classification system in which a primary catchment is the major unit [20]. A total of 11 hillslope seep wetlands were selected for the study; these are the dominant type of wetland in the catchment. The selection of the wetlands took into account biophysical factors such as slope aspect, soils, and geological characteristics such as sedimentary shale, mudstone and sandstone, as well as the degree of erosion, which was visually assessed (Table 1) [21]. In the T35D quaternary, three less eroded (LE1, LE2, LE3) wetlands were selected in an area recently changed from communal to private ownership. Eight wetlands were selected in T35E, of which four were moderately eroded (ME1, ME2, ME3, ME4) and four were highly eroded (HE1, HE2, HE3, HE4). These wetlands are situated in a communal area where there are no grazing management strategies [21]. The initial idea was to select 12 seep wetlands, but the level of degradation of wetlands in the catchment made it possible to find only three less eroded sites.

**Table 1:** Visual method used for estimating the degree of erosion of the studied hillslope seep wetland in the current study adopted from [25].

Erosional category	Description
**Low**	A few shallow (<0.5 m depth) gullies affecting no more than 5% of the surface; vegetation cover is good with little soil exposure.
**Moderate**	Presence of shallow to moderately deep gullies (0.5–1.0 m depth) and/or gullies affecting 5%–25% of the surface area; plant cover is moderate with small bare patches.
**High**	Presence of deep gullies (>1 m depth) and/or gullies affecting >25% of the surface; plant cover is very sparse with large bare areas.

Rural communities in the catchment rely heavily on natural resources and practise subsistence farming, which includes both livestock and crop production [22]. Overgrazing is an issue, with 70% of the catchment area under communal land tenure characterised by poor land management practice [22]. The average rainfall varies from 650 mm to 1000 mm per annum [22]. Temperatures range from an average of 14 ^o^C in winter to an average of 25 ^o^C in summer [23]. The area consists of mudstone, shale, and sandstone, with basalt material in the upper alpine zone. The mean elevation ranges from 1138–1243 m. The siliceous dispersive nature of the soils makes them highly erodible, increasing the susceptibility of hillslope seep wetlands to gully erosion. Of the 11 wetlands, five were seasonally, four were permanently and two were temporarily saturated wetlands. Seasonally saturated wetlands were dominated by a mixture of grasses, forbs and sedges, while temporarily saturated wetlands were dominated by grasses, and permanently saturated wetlands were dominated by sedges. Vegetation in the catchment is classified as sub-escarpment grassland and sub-escarpment savanna, dominated by moist grasslands and *Acacia* spp [24].

## Methods

### Vegetation sampling

Prior to data collection, traditional leaders were contacted to discuss the intended research, to request permission to use sites, to give clarity about the survey, and to make appointments for the interviews. Ethical clearance was obtained from the Rhodes University Ethics Committee. The consent was written and signed by traditional leaders

The vegetation of the 11 hillslope seep wetlands was sampled in summer (February) 2016 and winter (August) 2017 to assess the wetland conditions in the two seasons. A 100 m transect was established at the centre of each seep wetland in order to avoid the possibility of sampling terrestrial plant species. The very small sizes of hillslope seep wetlands render it important to avoid sampling terrestrial plant species [21]. Each transect was marked with small steel pegs so that they could be accurately located in the next sampling season. The vegetation in each site was sampled in two ways: first, the vegetation collection and cover were carried out using a quadrat method. Five quadrats (0.2 x 1 m) were placed along each transect at intervals of 20, 40, 60, 80, and 100 m. In each 0.2 x 1 m quadrat, species-relative cover and total vegetation cover were recorded. Secondly, all the vascular species were recorded along the transect to determine species composition using the step-point method [26].

### Indices used for assessing hillslope seep wetland condition

#### Floristic quality assessment index (FQAIall and FQAIdom)

A range of indices was used to assess the biological condition of the studied hillslope seep wetlands, after which the indices were evaluated for their performance. For each hillslope seep wetland, species were listed and assigned a CC, which is a subjective rating from 0 to 10. The CCs were assigned based on the opinion of a panel of four expert botanists. The original FQAI developed by [7,8] was modified to include non-native species which were taken as indicators of anthropogenic disturbance [27]. The FQAI was calculated based on both total (FQAIall) and dominant species (FQAIdom) for each site assessed. Dominant plant species in each site were defined as those whose cover was equal to or greater than 20% [28].

The CC scoring criteria in this paper was carried out following [29] and [30] as follows:

- 0–3: Plants with a broad range of ecological tolerance that are found in a variety of plant communities;
- 4–6: Plants with an intermediate range of ecological tolerance that are associated with a specific plant community;
- 7–8: Plants with a narrow range of ecological tolerance that are associated with advanced successional stage;
- 9–10: Plants with a high degree of fidelity to a narrow range of pristine habitats.

The FQAI score for each site was calculated using the following equation developed by [31]:

$$FQAI=\left(\frac{C}{10}\right)*\left(\frac{\sqrt{N}}{\sqrt{S}}\right)*100$$

where: C = Mean CC (determined by dividing the sum of the CC values of each species);

N = Native plant species richness; including both native and non-native species;

S = Total species richness at a site including non-native species.

#### Floristic Assessment Quotient for Wetlands (FAQWet index)

This index is based on wetness coefficients (WC) derived from the five main Wetland Indicator Categories given by [32] Each species is assigned a WC value from +5 (uplands) to -5 (obligates) (Table 2). The WC values for the present study were assigned based on the wetland plant species database and the wetland plant guide by [33]. The index is based on species richness, species endemism, and whether the species are more commonly found in wetland or non-wetland areas. A low score indicates low native species richness and/or non-wetland species; a high score indicates high native species richness and plants that are almost always found in wetlands [28]. The FAQWet scores for each site were calculated based on [11]:

FAQWet=∑WC/√S*N/S

where WC = the wetness coefficient value assigned to each species;

S = the species richness per site;

N = the number of native species at each site.

**Table 2:** Wetness coefficients based on wetland indicator status categories [11].

Indicator status	Probability of occurrence in wetlands	Wetness coefficient
Obligate wetland (OBL)	>99%	-5
FACW+		-4
Facultative Wetland (FACW)	67–99%	-3
FACW-		-2
FAC+		-1
Facultative (FAC)	34–66%	0
FAC-		+1
FACU+		+2
Facultative Upland (FACU)	1–33%	+3
FACU-		+4
Upland (UPL)	<1%	+5

#### The WET-Health assessment tool

This study employed WET-Health tool developed by [10] to assess the present state of the hillslope seep wetlands and identify the stressors contributing to their diminished health. A fieldwork assessment based on observed and measured attributes of each hillslope seep wetland was carried out to assess the present state, using three components at the hydrogeomorphic (HGM) unit level [10]. The WET-Health index uses three sub-metrics–hydrology, geomorphology and vegetation–to assess the present ecological status of a wetland. Each metric of the index is assessed and the score aggregated to provide an overall score reflecting the status of the site. A score ranging from 0–10 was calculated for each of the three component metrics, and the scores were placed into the following equation:

Health=[(Hydrologycategoryx3)+(Geomorphologycategoryx2)+(Vegetationcategoryx2)]/7

The WET-Health assessment is an impact–based approach that uses a scale of 0 to 10 with higher scores (8–10) indicating critically impacted, and lower scores (0–0.9) indicating a small impact or natural condition [10] (Table 3). The WET-Health assessment was only conducted in winter as it is based on the catchment characteristics, which are prone to fewer changes than site level characteristics that can easily change, given the size of hillslope seep wetlands. Despite being rapid, WET-Health assessment provides a more in-depth analysis by exploring hydrological, geomorphological and vegetation components of each hillslope seep wetland. It incorporates analysis at a catchment scale, a larger scale at which to look at external factors that affect the health of the wetland indirectly. In the present study, WET-Health was used to assess wetland condition as well as the stressors in assessing the performance of the response indices.

**Table 3:** Relationship between impact scores and present state of wetland condition [10].

Impact category	Description	Impact score range	Present state category
None	Unmodified, natural.	0–0.9	A
Small	Largely natural with few modifications in ecosystem processes and a small loss of natural habitats and biota.	1–1.9	B
Moderate	Moderately modified with moderate change in ecosystem processes; loss of natural habitats has taken place, but natural habitat remains predominantly intact.	2–3.9	C
Large	Largely modified with a large change in ecosystem processes; loss of natural habitat and biota.	4–5.9	D
Serious	The change in ecosystem processes and loss of natural habitat and biota is great, but some remaining natural habitat features are still recognisable.	6–7.9	E
Critical	Modifications have reached a critical level and the ecosystem processes have been modified completely; almost complete loss of natural habitat and biota.	8–10	F

### Assessing anthropogenic pressure in the studied hillslope seep wetlands

The AAI is as an index for qualitatively assessing the degree of human disturbance, based on visual inspection of a site [28]. The AAI was used to assess the degree of disturbance at each of the hillslope seep wetlands, using the AAI protocol developed by [11], who modified the index used by the Minnesota Department of Environmental Quality [34]. The AAI protocol includes some sections from the Rapid Assessment Method (RAM), the USA disturbance ranking system [35], and is based on the premise that human disturbances contribute to the degradation of wetland conditions [36]. Human disturbances at each studied wetland site were scored on five influence metrics: (i) surrounding land use intensity; (ii) soil disturbance; (iii) hydrological alteration; (iv) habitat alteration within the wetland; (v) vegetation community quality (Table 4).

**Table 4:** Anthropogenic Activity Index (AAI) for the Tsitsa River catchment, adapted from [11].

	**METRIC 1: Surrounding land use intensity**	
Degree of intensity	Description	Rating
Low	Mostly undisturbed but some human/animal influence (e.g., few livestock trails and footpaths).	1
Moderate	Moderate evidence of human/animal influence (e.g., active livestock grazing and/or small-scale agriculture).	2
High	Extensive evidence of human influence (e.g., commercial or large-scale farming (plantations)).	3
	**METRIC 2: Soil disturbance**	
Degree of disturbance	Description	Rating
Low	Small areas of bare soil (e.g., patches of soil and vegetation).	1
Moderate	Moderate areas of bare soil and/or desiccated soil (e.g., cracks in the soil).	2
High	Extensive areas of soil disturbance (e.g., gullies, rills and compacted soil).	3
	**METRIC 3: Hydrologic alteration**	
Degree of alteration	Description	Rating
Low	Low-intensity alteration (not currently affecting wetland).	1
Moderate	Significant and visible influence that is current and active.	2
High	High-intensity activity with major disturbance currently and actively affecting hydrology (e.g., ditch inlet, installed weir, levee, drainage channels, road bed, excavation, trampling, cultivation, dead vegetation, and others).	3
	**METRIC 4: Habitat alteration within the wetland**	
Degree of alteration	Description	Rating
Low	Some removal of vegetation, but vegetation is able to recover.	1
Moderate	Significant alteration (e.g., trampling, grazing and/or footpaths).	2
High	Intensive disturbance (e.g., overgrazing, trampling, bare soil).	3
	**METRIC 5: Vegetation community quality**	
Vegetation Quality	Description	
High	High species diversity and a predominance of native species, with non-native species absent or virtually absent.	1
Moderate	Moderate to moderately high species diversity and a predominance of native species, although non-native or disturbance-tolerant species may be present.	2
Low	Low species diversity and/or predominance of non-native or disturbance-tolerant native species.	3

Because the AAI disturbance criteria are subjectively assessed, two assessors undertook the exercise in the field and their results were compared and harmonised. The AAI ranges from 1 to 15 (Table 4). Wetlands with scores 1 to 5 are regarded as least disturbed; 6 to 10 moderately disturbed, and >10 highly disturbed. Scores from the five metrics are summed to obtain the degree of disturbance per site per season.

### Statistical analysis

#### Evaluating the performance of FQAIall, FQAIdom, and FAQWet for assessing hillslope seep wetland health

In order to evaluate the performance of the studied indices in relation to hillslope seep wetland conditions, the indices, that is, FQAIall, FQAIdom, and FAQWet, were regressed against AAI and WET-Health. Both AAI and WET-Health were used as a measure of stressors contributing to disturbance. The significance of the correlation was assessed at p ≤ 0.05. The linear regression analyses were undertaken using R version 3.4.0.

#### Assessing the redundancy between FQAIall and FQAIdom

The redundancy between FQAIall and FQAIdom was tested using Spearman’s rank correlation analysis. The redundancy between the two indices was assessed in order to ascertain whether they can be used interchangeably, particularly because FQAIall usually demands CC value for all species, which may not always be available. Spearman’s rank correlation analyses were run in STATISTICA version 13.3.

## Results

### Plant species assemblage structure, the coefficient of conservatism and indicator status

A total of 78 species were identified over the study period. Of these, 52% were recorded in summer, 17% in winter, and 31% in both summer and winter. Across all the sites, the majority of the species identified were facultative upland and obligate wetland species, with the highest percentages of 33%, and 27%, respectively (Table 5). A high number of sensitive species, such as *Kyllinga erecta*, *Themeda triandra*, *Tristachya hispida* with high CC scores (9–10) were observed in summer. These species are less tolerant of ecological disturbance and are considered to be restricted to largely unimpacted areas. However, the majority of the species observed in winter were those with a high and moderate range of tolerance of ecological disturbance e.g., *Stenotaphrum secundatum*, *Cynodon dactylon*, *Verbena brasiliensis* (Table 5). Among the species recorded in summer, the dominant ones were *Cymbopogon validus*, *Cyperus denudatus*, *Cyperus longus*, *Digitaria erientha*, *Eragrostis curvula*, *Eragrostis plana*, *Haplocarpa lyrata*, *Helichrysum aureonitens*, *Hemarthria altissima*, *Juncus acutus*, *Mentha aquatic*, *Miscanthus capensis*, *Paspalum distichum*, *Scirpus nodosus*, *Senecio coronatus*, *Sporobolus Africanus*, and *Richardia brasiliensis*. The dominant species in winter were *Centella asiatica*, *Cymbopogon validus*, *Cyperus congestus*, *Cyperus longus*, *Eragrostis curvula*, *Eragrostis plana*, *Helichrysum nudifolium*, *Hyparrhenia dregeana*, *Juncus effusus*, *Marsilea minuta*, *Miscanthus capensis*, *Paspalum distichum*, *Paspalum dilatatum*, and *Cynadon dactylon*.

**Table 5:** Plant species present in all study sites with their assigned coefficient of conservatism and wetland indicator status. Species marked with superscript (a) are dominant, (x) indicates species occurrence.

Plant Species	Seasons		CC
Indicator status	Winter/Summer		
**Obligates**			
*Callitriche* spp		x	5
*Cyperus denudatus*^*a*^		x	7
*Cyperus fastigiatus*		x	7
*Cyperus longus*^*a*^	x	x	6
*Cyperus marginatus*		x	6
*Ficinia* spp		x	0
*Ficinia nodosa*		x	0
*Fimbristylis complanata*		x	6
*Hemarthria altissima*^*a*^		x	7
*Isolepis fluitans*	x		8
*Juncus dregeanus*		x	8
*Juncus effusus*^*a*^	x	x	4
*Juncus lomatophyllus*		x	4
*Kniphofia* spp		x	8
*Kyllinga erecta*		x	9
*Marsilea minuta*^*a*^	x		6
*Mentha aquatica*^*a*^		x	7
*Paspalum distichum*^*a*^	x	x	3
*Knowltonia bracteata*		x	7
*Phragmites australis*		x	6
*Scirpus nodosus*^*a*^		x	7
**Facultative Wetland (FACW)**			
*Commelina africana*		x	5
*Cyperus congestus*^*a*^	x	x	5
*Helichrysum aureonitens*^*a*^	x	x	6
*Helichrysum mundtii*		x	7
*Juncus acutus*^*a*^	x	x	5
*Miscanthus capensis*^*a*^	x	x	8
*Panicum maximum*	x	x	3
*Paspalum dilatatum*	x	x	0
*schizachyrium sanguineum*		x	1
*sporobolus fimbriatus*	x	x	6
**Facultative (FAC)**			
*Hyperrhia hirta*	x		5
*Kyllinga alata*		x	9
*Polygonum* spp	x	x	5
*Sporobolus africanus*^*a*^	x	x	4
*Richardia humistrata*	x	x	0
*cp Ajuga ophrydis*		x	9
*Trifolium repens*		x	0
**Facultative upland species (FACU)**			
*Alepidea amatymbica*	x	x	9
*Alloteropsis semialata*		x	8
*Berkheya* spp		x	3
*Centella asiatica*^*a*^	x	x	0
*Conyza scabrida*		x	5
*Cynadon dactylon*	x	x	3
*Digitaria erientha*^*a*^		x	5
*Eragrostis capensis*		x	5
*Eragrostis curvula*^*a*^	x	x	5
*Eragrostis plana*^*a*^	x	x	4
*Eragrostis planiculmis*	x		6
*Haplocarpa lyrata*^*a*^		x	8
*Hyparrhenia dregeana*^*a*^	x	x	5
*Hypoxis acuminata*		x	7
*Hypoxis colchicifolia*	x		7
*Hypoxis* spp		x	7
*Lithospermum papillosum*	x		6
*Richardia brasiliensis*		x	0
*Senecio coronatus*^*a*^		x	6
*Senecio spp*		x	3
*Senecio speciosus*		x	6
*Stenotaphrum secundatum*	x		0
*Gerbera viridifolia*		x	6
*Verbena brasiliensis*	x		0
*Wahlenbergia* spp		x	8
**Upland (UP)**			
*Argyrolobium stipulaceum*		x	7
*Baleria* spp	x		3
*Cheilanthes hirta*		x	8
*Corchorus asplenifolius*	x		6
*Cymbopogon plurinodis*	x		7
*Cymbopogon validus*^*a*^	x	x	7
*Eragrostis aspera*		x	2
*Erigeron karvinskianus*	x		0
*Geranium sanguineum*	x	x	6
*Helichrysum nudifolium*^*a*^	x	x	6
*Ornithogalum* spp		x	1
*Senecio inaequidens*	x		6
*Taraxicum officinale*	x	x	0
*Themeda triandra*	x	x	9
*Tristachya hispida*		x	9

#### Assessing the conditions of the hillslope seep using WET-Health, FQAI and FAQWet

**Fig 2:**
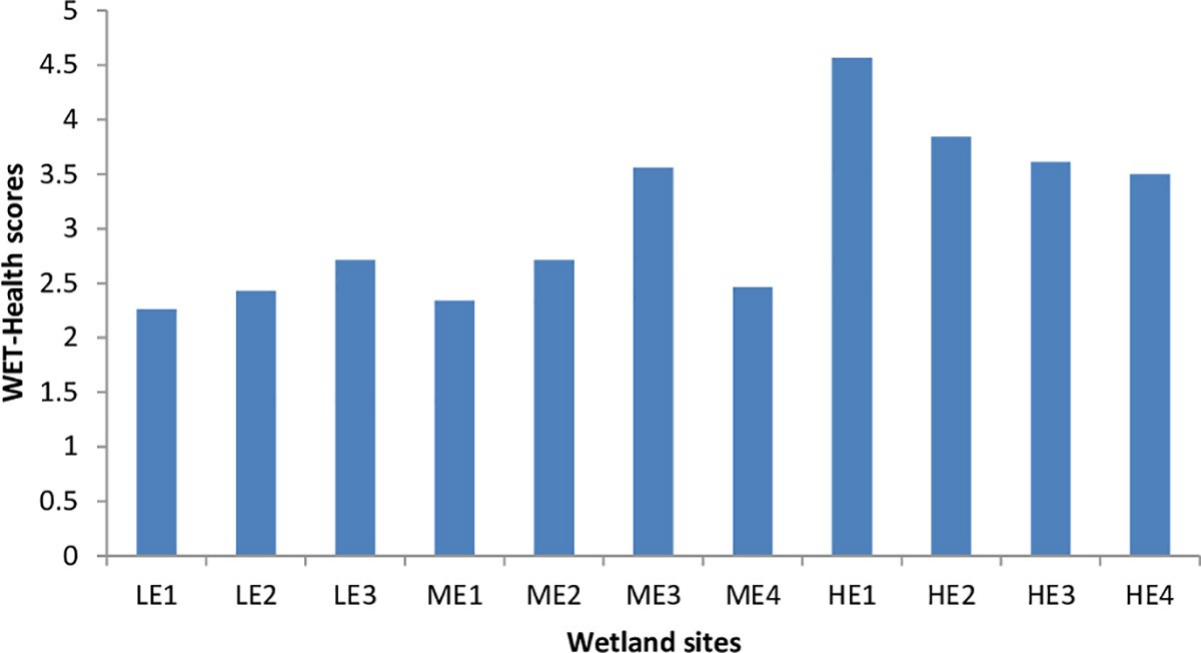
The WET-Health scores recorded in each of the selected wetland sites (LE 1–3 = Less Eroded, ME1-4 = Moderately Eroded, HE1-4 = Highly Eroded).

WET-Health results showed that the present state of ten out of eleven wetlands were assessed as category C, signifying that these hillslope seep wetlands had undergone moderate modifications. Only one site (HE1), with the highest impact score of 4.57, was categorised as a category D present state, which signified that the wetland had undergone large modifications in ecosystem processes and habitat loss. Although ten wetlands were assessed as category C, less eroded sites had lower scores than the highly eroded sites (Fig 2).

**Fig 3:**
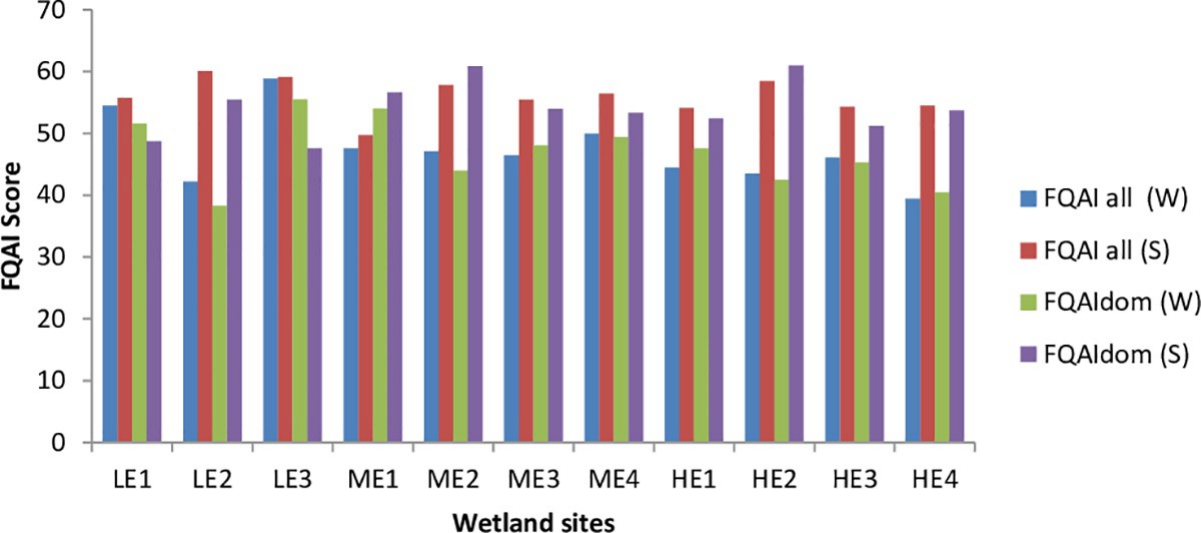
Floristic Quality Assessment Index (FQAI recorded in each of the selected wetlands in two seasons. (LE 1–3 = Less Eroded, ME1–4 = Moderately Eroded, HE1–4 = Highly Eroded).

**Fig 4:**
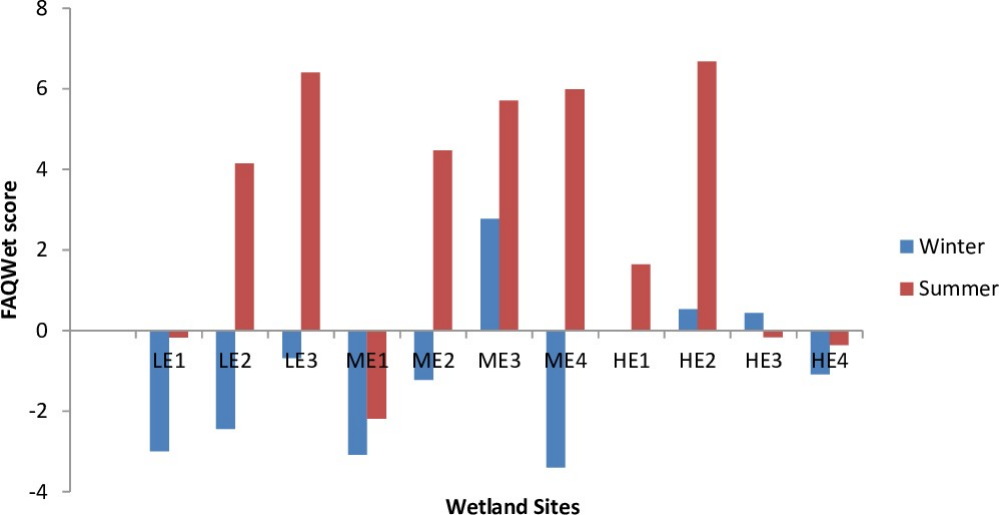
FAQWet scores recorded in each of the selected wetland sites and seasons. (LE 1–3 = Less Eroded, ME1–4 = Moderately Eroded, HE1–4 = Highly Eroded)

The FQAI (all and dom) scores were lower in winter than in the summer season (Fig 3). The majority of sites in winter had FQAI scores of 38 to 45, while the majority of sites in the summer season had FQAI scores of 55 to 60. Comparison of the results of sites in winter showed that the less eroded sites had higher scores than the highly eroded sites. The less eroded sites are in a privately owned area with less grazing pressure, while highly eroded sites are in communal grazing land with open access for grazing. However, FQAI scores during the summer season show little difference among the sites. Examination of the FAQWet index show that the sites in summer had FAQWet scores close to 6, which were higher than the winter site scores of close to -3 (Fig 4). A low FAQWet score indicates low native species richness and/or non-wetland species, while a high score indicates high native species richness and plants that are almost always found in wetlands.

#### Evaluating the performance of FQAIall, FQAIdom FAQWet, in relation to AAI and WET-Health

**Fig 5:**
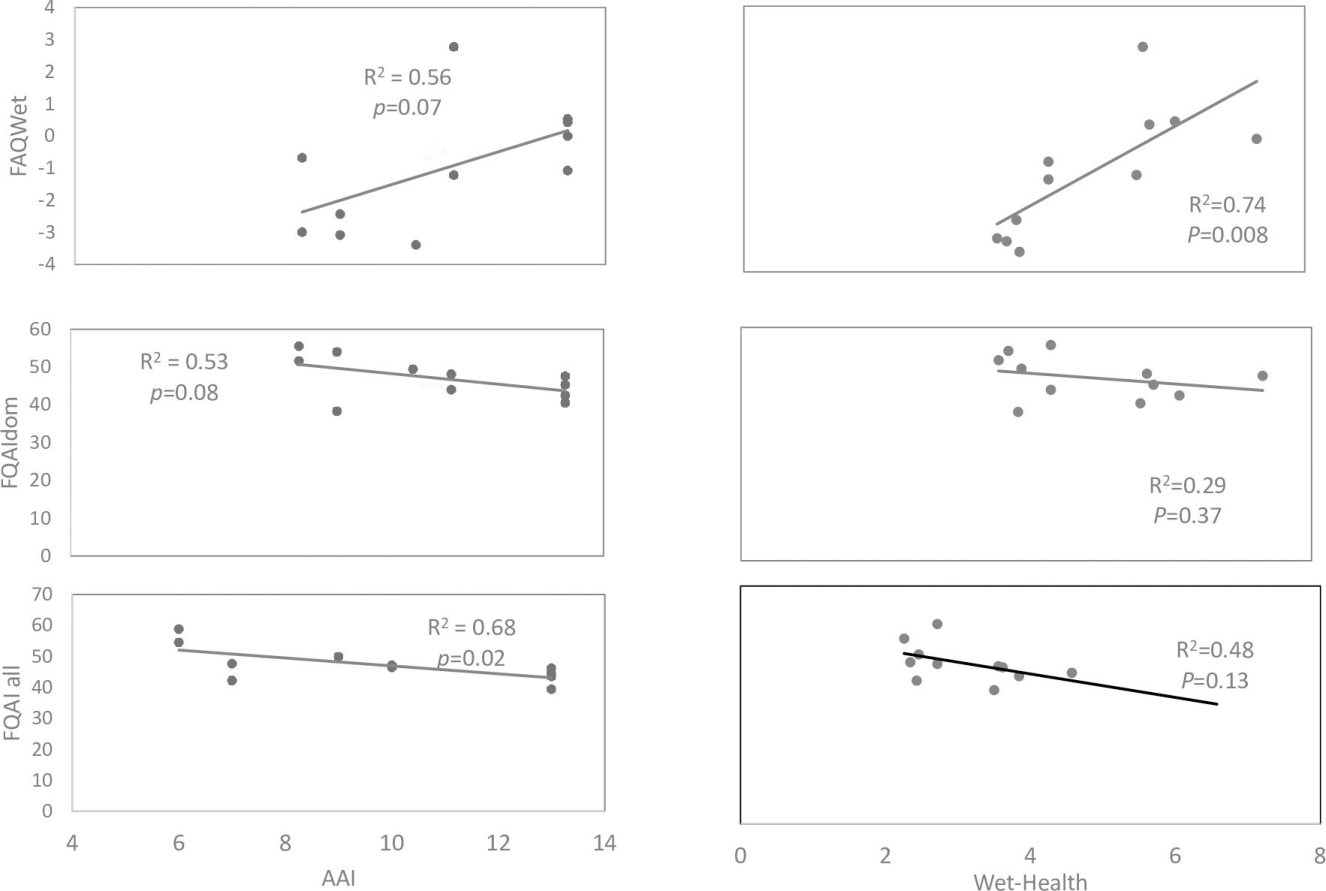
Comparison of linear regression analyses for FQAIall, FQAIdom, and FAQWet with AAI and WET-Health, for winter season for the 11 surveyed wetland sites.

Linear regression analysis was used to assess the performance of the three indices and evaluated separately for the winter and summer seasons. In winter, all indices–FQAIall, FQAIdom, and FAQWet–were significantly related to AAI. The response of FQAIall to AAI was stronger (R^2^ = 0.68, p = 0.021) than that of FQAIdom to AAI (R^2^ = 0.53, p = 0.08) and FAQWet (R^2^ = 0.56, p = 0.07) (Fig 5). Both FQAIdom and FAQWet showed a moderate but not significant relationship with AAI (Fig 5).

**Fig 6:**
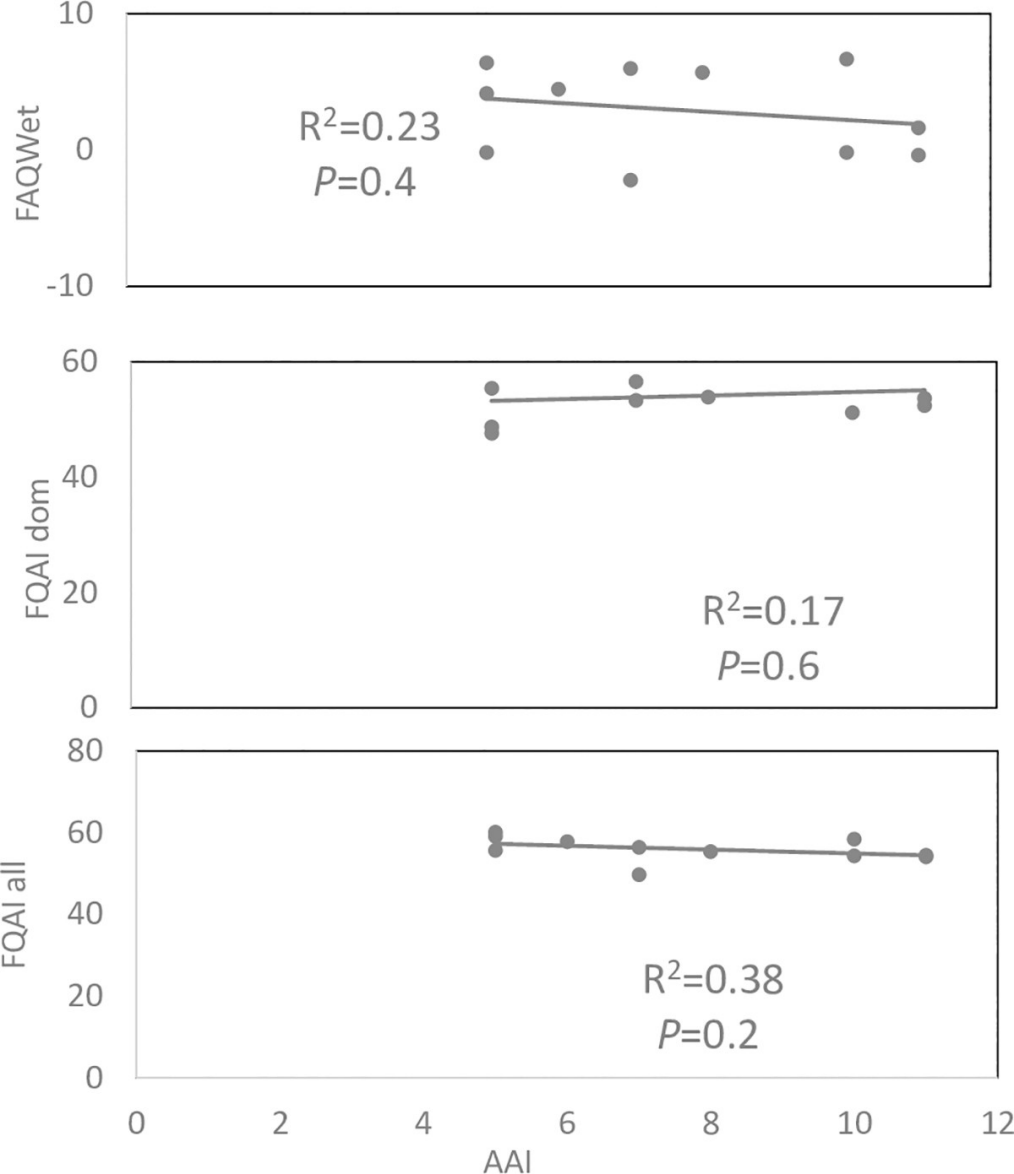
Comparison of linear regression analyses for FQAIall, FQAIdom, and FAQWet with AAI, for summer season for the 11 surveyed wetland sites.

When comparing all indices with WET-Health, FAQWet had a stronger significant relationship with WET-Health (R^2^ = 0.74, p = 0.008), than that of FQAIall (R^2^ = 0.48, p = 0.13) and FQAIdom (R^2^ = 0.29, p = 0.37). FQAIall showed a moderate relationship, while FQAIdom showed a weak relationship that was not significant with WET-Health (Fig 5). In summer, all assessed indices–FQAIall, FQAIdom, and FAQWet–showed weak relationships with AAI that were not significant (Fig 6).

#### Evaluating redundancy (co-linearity) between FQAIall and FQAIdom

**Fig 7:**
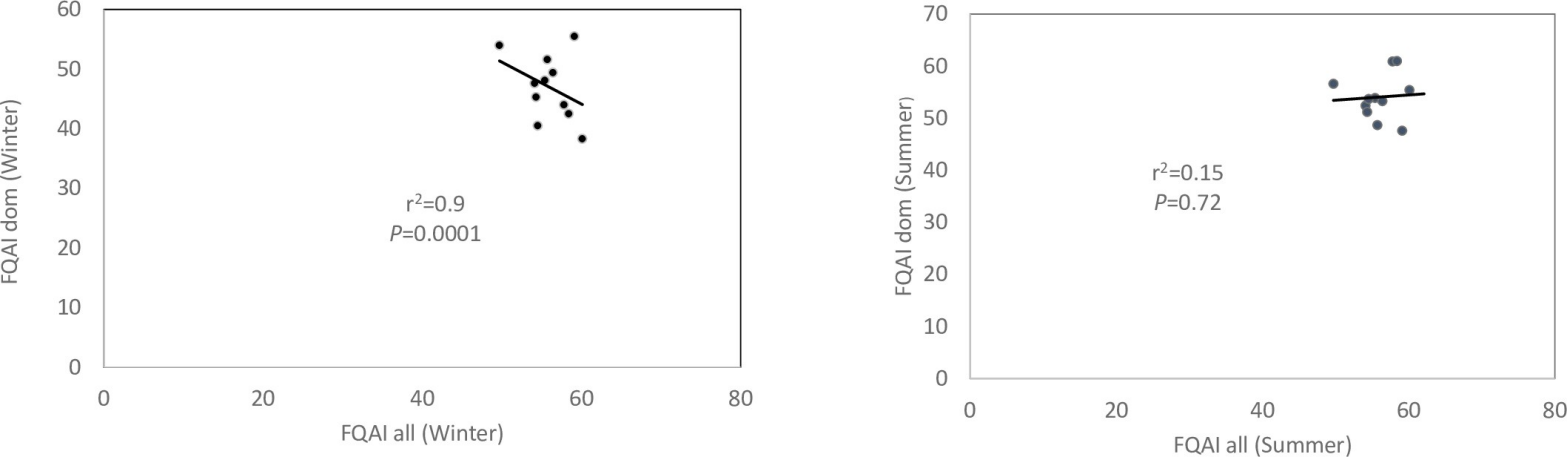
Regression analysis of FQAIall (all species) and FQAIdom (dominant species) for summer and winter.

Spearman’s correlation was run to assess the correlation between FQAIall and FQAIdom. The Spearman’s correlation results showed a strong positive correlation between the two indices in winter (r = 0.9, *p* = 0.0001) (Fig 7), indicating that the two indices were highly redundant in winter. In summer, there was no significant correlation (r = 0.15, *p* = 0.72) between the two indices (Fig 7) indicating non-redundancy between the two indices. Overall, the result suggests that seasonality plays a significant role in terms of whether FQAIall or FQAIdom is used, as the two indices were highly redundant in winter, with no redundancy in summer.

## Discussion

### Plant species assemblage structure

Plants are regarded as good indicators of wetland condition because some species often have a rapid growth rates, and respond quickly to ecological changes [37]. The number of species recorded in the present study was similar to that reported by [38] who conducted a floristic composition in the grassland of the same catchment. The results of this study indicate more sensitive species were identified in summer than in winter. These species include *Kyllinga erecta*, *Themeda triandra*, and *Tristachya hispida* which are preferred by grazing animals because of their palatability. The dominance of sensitive species with high CC (e.g., 8) in summer rather than in winter suggests that grazing pressure increased in hillslope seep wetlands during the winter. The increase in grazing intensity in winter is driven by lack of fresh green vegetation in the surrounding rangeland, and the grazing pressure has led to the seasonal decline of sensitive species which are likely to be palatable. A study conducted by [39] found a similar shift in species composition in the vegetation as a result of high grazing pressure in the dry season.

### Assessing the ecological condition of hillslope seep and the performance of FQAIall, FQAIdom, and FAQWet

Hillslope seep wetlands are critical ecosystems in the Tsitsa catchment because of their potential for supplying vegetation for all-year-round grazing. From a biophysical perspective, they are unique because of their small size, their great dependence on groundwater, their location on steep slopes, and their evergreen nature within the context of the broader catchment. Despite these unique features, existing indices have not been applied specifically to hillslope seep wetlands. The present study combined widely used wetland indices FQAIall, FQAIdom, and FAQWet to assess the conditions of hillslope seep wetlands and to assess the indices’ performance using WET-Health and AAI indices.

Based on the WET-Health index, the results showed that the majority of hillslope seep wetlands were in category C, suggesting that the wetlands have been moderately impacted, chiefly by agriculture activities. Similar results were reported by [40] who reported that the major impacts on hillslope seep wetlands stem from overgrazing and trampling by livestock.

The FQAI and FAQWet results also indicated that hillslope seep wetlands in communal areas were more degraded than those in privately owned lands. The degradation of hillslope seep wetlands in communal areas could be attributed to poor wetland management practices such as intense livestock grazing, and alien invasive species around the communal wetlands [2]. Similarly, a study conducted by [41] in the Free State, South Africa, comparing the ecological status of wetlands in communal and on private commercial farms found that communal wetlands were generally in a poor state as a result of uncontrolled livestock grazing [42]. [43] reported hillslope seeps as favoured foraging and drinking areas for livestock in communal areas and found that seep wetlands had three times more bare ground in communal areas than in areas under conservation.

Although all three indices used in this study indicated that hillslope seep wetland had been impacted, FQAIall performed better than FQAIdom and FAQWet when regressed against AAI, while FAQWet performed better than FQAIall and FQAIdom when regressed with WET-Health [44] indicated that, by nature, many rapid indices such as FQAIdom would exclude rare species, therefore the low performance of FQAIdom could be attributed to eliminating rare species that might have had negative consequences for wetland condition. [16], also suggested that targeting only abundant species introduces uncertainty related to intra- and inter-annual variability, and this approach need to be adopted with caution.

Given that the studied wetlands are impacted by disturbances such as grazing, indices using CC scoring criteria would give better ecological condition results because CC are based on the tolerance of a species to disturbances. This could explain why FAQWet performed worse than the FQAI when regressed with AAI because it is based on WC and species richness, which are less directly related to disturbances. [28] also found that FQAI responded more strongly to anthropogenic activities than the FAQWet. Although a study conducted by [11] found that the FAQWet method performed as well as the widely accepted FQAI across a broad gradient of human activity, they also found that the FQAI and disturbance correlation was stronger than the FAQWet.

The stronger response of FAQWet to WET-Health compared to AAI observed in the present study could be attributed to the fact that, unlike the other tools, the FAQWet index assesses vegetation changes that are influenced by hydrological processes to indicate the level of wetness in a wetland, and the overall score of WET-Health is based on three environmental components: hydrology, geomorphology and vegetation. The hydrology component of WET-Health includes more indicators and more detailed prescriptions in terms of determining scores than AAI does.

The FQAI and FAQWet results provided evidence that degradation was more pronounced during the winter season than the summer season. The evidence of degradation in winter could be attributed to high grazing intensity that led to lower FQAI and a low number of obligate specie that produced low FAQWet scores during the dry season. A number of studies [45–46] have investigated the inter-annual and seasonal variability of FQAI scores. In the present study, high FQAI scores during the summer season could be explained by the high species richness of *Kyllinga erecta*, *Themeda triandra*, and *Tristachya hispida*, all of which were allocated the highest CC score. Although, in the present study, seasonal variability was observed for the FQAI score, [45] found little difference in FQAI scores across years, the same study also indicated that variation of FQAI scores across years could originate from fluctuations in species composition and that changes in disturbance regimes led to the invasion and establishment of exotic species which could decrease FQAI scores.

### Assessing the redundancy between FQAIall and FQAIdom

The study by [12] indicated that neglecting species, either unintentionally or through the inability to identify taxa to species level, or deliberately using only dominant species, may be of little consequence to the overall assessment results. However, in the present study, when the Spearman rank correlation between FQAIall and FQAIdom was undertaken, the overall result suggested that seasonality plays a significant role in terms of whether FQAIall or FQAIdom can be used interchangeably. The winter results produced high redundancy between the two indices, indicating that there might be minimal consequences in using only dominant species. In summer, however, minimal redundancy was observed between the two indices. The results of this study imply that assessing the ecological health of hillslope seep wetland, particularly using dominant species in the summer season, might provide insufficient insight into wetland condition. Therefore, seasonality is crucial in assessing the ecological condition of hillslope seep wetlands when deciding whether to use FQAIall or FQAIdom.

## Conclusion

All assessed indices showed that hillslope seep wetlands have been modified by anthropogenic disturbances. By comparing the responses of FQAIall, FQAIdom and FAQWet to AAI and WET-Health, the current study provides evidence for the potential use of FQAIall and FAQWet in wetland condition assessment. A stronger relationship between FQAIall and AAI, and that of FAQWet and WET-Health showed that these are potentially useful tools for assessing the ecological condition of hillslope seep wetland ecosystems. In South Africa, WET-Health is the primary tool used to assess wetland condition, but no studies have used WET-Health performance with tools such as FQAI and FAQWet, that are used elsewhere. The present study, therefore, provides evidence for the use of FQAIall and FAQWet in assessing the health of hillslope seep wetlands. A key limitation of the present study is that South Africa has no comprehensive list of species with assigned CC scores, and research is needed to compile a database of regional wetland plant species with their coefficients of conservatism. Unfortunately, the limitation could not be circumvented through the use of only dominant species as the current results indicated that FQAIall and FQAIdom were not redundant during the summer season. Another limitation is the low resolution at which disturbances were assessed and the high level of subjectivity required to make such assessment. A possible area for future research would be to identify wetland sites with known disturbance regimes which require less subjective appraisal.
